# Research note: Effects of dietary supplementation of *Abelmoschus manihot* L. flower extract on growth performance, antioxidant capacity, intestinal health, and welfare in broiler chickens

**DOI:** 10.1016/j.psj.2025.106358

**Published:** 2025-12-29

**Authors:** Gyu Lim Yeom, Ha Neul Lee, Yeong Bin Kim, Ju Yeong Park, Geun Yong Park, Ji Won Shin, Sanghun Park, Gyutae Park, Soyoung Jang, Yang-il Choi, Jungseok Choi, Jong Hyuk Kim

**Affiliations:** a Department of Animal Science, Chungbuk National University, Cheongju 28644, South Korea; b Research Center, Orge CO., LTD., Jecheon 27157, South Korea

**Keywords:** *Abelmoschus manihot* L., Broiler chicken, Flower extract, Growth performance, Welfare

## Abstract

The objective of this experiment was to investigate the effects of dietary supplementation of *Abelmoschus manihot* (AE) L. flower extract on growth performance, antioxidant capacity, intestinal health, and welfare in broiler chickens. A total of 144 one-d-old Arbor Acres broiler chickens were allotted to 1 of 3 dietary treatments with 8 replicates per treatment in a completely randomized design. Each replicate consisted of 6 birds. The corn-soybean meal-based basal diet was formulated without supplemental AE. Two Additional diets were prepared by adding AE to the basal diet at inclusion levels of 0.02 or 0.04%. The experimental diets were fed to birds for 35 d. Results indicated that birds fed diets containing 0.04% AE had greater (*P* < 0.05) feed efficiency (FE) than those fed the basal diet. Body weight (BW), BW gain, and FE of broiler chickens increased (linear, *P* < 0.05) as inclusion levels of AE in diets increased. Feeding diets supplemented with 0.04% AE showed greater (*P* < 0.05) villus height to crypt depth (VH:CD) ratio than feeding diets supplemented with 0 and 0.02% AE. Increasing inclusion levels of AE in diets decreased (linear, *P* < 0.05) CD and increased (linear, *P* < 0.05) VH:CD ratio. Feeding diets supplemented with 0.04% AE showed less (*P* < 0.05) gait score than feeding diets supplemented with 0% AE, and gait score decreased (linear, *P* < 0.05) by increasing inclusion levels of AE in diets. Collectively, dietary AE supplementation showed positive effects on growth performance and intestinal morphology, without adverse impacts on hepatic antioxidant capacity or welfare parameters in broiler chickens. These results indicate that AE may be considered a promising phytogenic feed additive in broiler diets to improve productivity and intestinal health of broiler chickens.

## Introduction

In recent decades, global demand for poultry meat has risen sharply, fueled by increasing incomes, rapid urbanization, and a greater awareness of the nutritional value of animal-derived proteins. To address this demand, enhancing poultry productivity through growth-promoting substances is considered crucial. Historically, antibiotic growth promoters (AGPs) were extensively used for this objective. However, concerns regarding the presence of antibiotic residues in meat and the development of antibiotic-resistant bacteria in humans have led to significant public health concerns. Consequently, the European Union enforced a ban on AGPs in 2006, leading to widespread research into natural feed additives with similar efficacy and minimal negative effects. However, the sudden withdrawal of AGPs from poultry feed without efficient replacements can negatively impact poultry health and welfare. As a consequence, a range of natural feed additives including probiotics, prebiotics, organic acids, enzymes, and phytogenic feed additives (PFAs) have been evaluated as substitutes for AGPs (Ayalew et al., 2022).

Among these alternatives, PFAs are natural compounds sourced from herbs, spices, and other plant-based materials and have garnered significant interest. These additives are recognized for their ability to stimulate appetite and feed intake (FI), boost digestive enzyme activity, and enhance antioxidant activity, thereby supporting intestinal health and overall poultry productivity. Furthermore, recent studies have demonstrated that antioxidant-rich PFAs may reduce oxidative stress, reinforce immune function, improve nutrient utilization, and modulate intestinal function in poultry. Moreover, supplementing broiler diets with PFAs has also been reported to enhance carcass quality (Swelum et al., 2021).

*Abelmoschus manihot* L. is a significant plant that has been traditionally consumed throughout East and Southeast Asia, including China, Taiwan, and Indonesia, owing to its nutritional and medicinal properties. The flowers of *Abelmoschus manihot* L. contain abundant bioactive constituents such as flavonoids, polysaccharides, organic acids, and phenolic compounds, which demonstrate antioxidant, anti-inflammatory, and antimicrobial activities (Todarwal et al., 2011). These activities indicate its potential value as a natural PFAs in poultry feed. Importantly, the flower extract is regarded as the most pharmacologically active form due to its elevated levels of functional bioactive compounds, thereby potentially improving animal health and welfare more efficiently than other parts of the plant. Although previous studies have examined *Abelmoschus* species in poultry, most have used non-extracted plant materials, particularly leaf or seed powders, at relatively high dietary inclusion levels. In contrast, the present study is the first to evaluate a flower-derived extract of *Abelmoschus manihot* as a low-level dietary phytogenic additive in broiler chickens. Based on the known bioactive properties of *Abelmoschus manihot*, we hypothesized that dietary supplementation with its flower extract would influence growth performance and physiological responses in broiler chickens, and that such effects would indicate its potential as a phytogenic feed additive.

Therefore, this study aimed to evaluate the effects of dietary supplementation of AE on growth performance, antioxidant capacity, intestinal health, and welfare in broiler chickens.

## Materials and methods

### Sample preparation

*Abelmoschus manihot* L. flowers were freshly sourced from a local farm (Kounblack, Goesan-gun, Chungcheongbukdo, Republic of Korea). Following oven-drying, the flowers were finely ground to a particle size below 1 mm and stored in a refrigerator. To prepare the extract, the dry powder was mixed with water at room temperature to reach a material-to-liquid ratio of 1:10 (w/v) for 24 h. The resulting, the mixture was concentrated at 60°C under reduced pressure with an extractor (COSMOS-660, Kyungseo E&P, Co., Ltd., Incheon, Republic of Korea) to produce an aqueous extract. The extract was subsequently freeze-dried to obtain powdered AE, which was then stored in a refrigerator.

### Animals, diets, and experimental design

The protocol for this experiment was reviewed and approved by the Institutional Animal Care and Use Committee at Chungbuk National University (CBNUA-2268-24-01). A total of 144 1-d-old mixed-sex Arbor Acres broiler chicks (72 males and 72 females) were obtained from a local hatchery (Dongsan Hatchery, Cheonan, Chungcheongnamdo, Republic of Korea). Chicks with an average body weight (BW) of 43.3 *g* ± 1.3 g were randomly allocated to 3 treatment groups, each containing 48 birds per group, and divided into 8 replicates of 6 birds per replicate, in a complete randomized design. Birds in each replicate were reared in single cage with four layers (52 × 61 × 42 cm = width × length × height) for 35 d. The experimental groups consisted of a basal diet, a basal diet supplemented with 0.02% AE, and a basal diet supplemented with 0.04% AE. The basal diet was formulated based on the nutritional guidelines recommended for broiler chickens in the Arbor Acres Broiler Nutrition Specifications (Aviagen, 2022). Details of the composition and nutrient content of the basal diet are provided in Table 1. Broiler chickens were fed either the basal diet or basal diet containing AE, both of which were provided in mash form during the grower (1 - 21 d) and finisher (22 - 35 d) phases. All diets and water were provided ad libitum for the duration of 35 d. The experimental temperature was maintained at 30°C during the first wk and gradually reduced to 24°C at the end of the experiment. The lighting schedule was set to 24 h throughout the experimental period. The BW gain (BWG) and FI were recorded at the end of the experiment. The feed efficiency (FE) was calculated by dividing BWG with FI.

**Table 1:** Composition and nutrient contents of the experimental diets (as-fed basis).

Ingredients	Grower (0 - 21 d), %	Finisher (22 - 35 d), %
Corn	56.46	63.24
Soybean meal, 46% CP	30.08	25.38
Corn gluten meal	6.65	5.00
Soybean oil	2.23	2.63
Salt	0.30	0.30
MCP	0.75	1.32
Limestone	1.14	0.84
98.5% DL-Met	0.28	0.26
55% l-Lys H_2_SO_4_	0.44	0.38
98.5% l-Thr	0.02	0.00
50% choline	0.10	0.10
NaHCO_3_	0.15	0.15
Vitamin premix^1^	0.20	0.20
Mineral premix^2^	0.20	0.20
Total	100.00	100.00
Calculated energy and nutrient contents^3^		
AME_n_, kcal/kg	3,013	3,100
CP, %	22.25	19.50
Digestible Lys, %	1.25	1.08
Digestible Met + Cys, %	0.96	0.86
Digestible Met, %	0.53	0.48
Digestible Thr, %	0.84	0.72
Digestible Trp, %	0.20	0.17
Total calcium, %	0.85	0.65
Available phosphorus, %	0.46	0.36

*Abbreviations*: MCP = monocalcium phosphate.

1 Provided per kilogram of the complete diet vitamin A (from vitamin A acetate), 12,000 IU; vitamin D_3_, 4,000 IU; vitamin E (from DL-α-tocopheryl acetate), 80 IU; vitamin K_3_, 4 mg; vitamin B_1_, 4 mg; vitamin B_2_, 10 mg; vitamin B_6_, 6 mg; vitamin B_12_, 20 μg; calcium pantothenate, 20 mg; folic acid, 2 mg; biotin, 200 μg; niacin, 60 mg.

2 Provided per kilogram of the complete diet: Zn (as ZnO), 85 mg; Mn (as MnSO_2_·H_2_O), 90 mg; Cu (as CuSO_4_·5H_2_O), 15 mg; Co (as CoCO_3_), 25 μg; I (as Ca (IO_3_)_2_·H_2_O), 1.5 mg; Se (as Na_2_SeO_3_), 25 μg.

3 Calculated values from the Arbor Acres broiler nutrition specifications (Aviagen, 2022).

### Sample collection

At the conclusion of the experiment (35 d of age), 1 male broiler chicken per replicate was selected based on a BW that closely matched the average BW within each replicate. Following CO_2_ asphyxiation, the liver samples were divided into small portions, placed into 1.7 mL microtubes and preserved at −80°C for later analysis of malondialdehyde (MDA), total antioxidant capacity (TAC), glutathione (GSH), catalase (CAT), and superoxide dismutase (SOD). Middle jejunum segments (2 - 3 cm) were collected and fixed in 10% neutral-buffered formalin solution for intestinal morphological examination.

### Antioxidant capacity

The levels of MDA, TAC, GSH, CAT, and SOD in liver tissue were quantified using commercial assay kits. Specifically, MDA levels were measured with the EZ-Lipid Peroxidation (TBARS) Assay kit (DG-TBA200, DoGenBio, Seoul, Republic of Korea) according to the manufacturer’s protocol. TAC levels were determined using the EZ-Total Antioxidant Capacity Assay Kit (DG-TAC200, DoGenBio, Seoul, Republic of Korea). The levels of GSH were evaluated using the EZ-Glutathione assay kit (DG-GLU200, DoGenBio, Seoul, Republic of Korea), the levels of CAT were assessed with the EZ-Catalase assay kit (DG-CAT400, DoGenBio, Seoul, Republic of Korea), and the levels of SOD were assessed with the EZ-SOD assay kit (DG-SOD400, DoGenBio, Seoul, Republic of Korea). All assays were conducted in strict accordance with the manufacturer’s instructions.

### Jejunal morphology

The fixed jejunal tissues were washed, dehydrated, cleared, and subsequently embedded in paraffin. Paraffin sections with a thickness of 4 μm were placed on glass slides for hematoxylin-eosin staining. Microscopic images of all slides were acquired at 40× magnification using a microscope equipped with a camera. Each analysis included measurements of 10 intact villi. Villus height (VH) was defined as the distance from the tip of the villus to the villus-crypt junction and crypt depth (CD) was measured from the crypt base to the transition region between crypt and villus. Villus width (VW) was assessed at the widest part of each villus, and VH:CD ratio was calculated. The villus surface area (VSA) was also calculated using the following formula:

VSA (mm^2^) = π × VW × VH

### Welfare assessment

At 35 d, 3 male birds per replicate were randomly selected for welfare evaluation. Welfare parameters in broiler chicken were assessed according to the Welfare Quality Assessment Protocol for Poultry (Welfare Quality, 2009). Parameters including gait score, footpad dermatitis, hock burn, and plumage cleanliness were recorded visually. All evaluations were performed by five investigators. The gait score ranged from 0 to 5, with 0 representing a normal gait and 5 indicating complete inability to stand or walk. Assessment of footpad dermatitis involved examination of both right and left footpads. Scores for footpad dermatitis ranged from 0 (no lesion) to 4 (severe lesion). Hock burn was graded based on the severity of inflammation as follows: 0, no lesion; 1 and 2, small and medium-sized inflammation; 3 and 4, large or ulcerated inflammation. Plumage cleanliness was scored from 0 to 3, with 0 indicating clean, white plumage with no fecal staining and 3 indicating dirty brown plumage, sometimes with feces adhering or encrusted to the feathers.

### Statistical analysis

All data were analyzed using a completely randomized design with the PROC MIXED procedure in SAS (SAS Institute Inc., Cary, NC). The experimental unit for all data analysis was the replicate. Outlier detection was performed with the UNIVARIATE procedure of SAS, and no outliers were identified. Treatment means were calculated using the LSMEANS procedure, and the PDIFF option of SAS was applied to separate means when differences were statistically significant. Orthogonal polynomial contrast tests evaluated linear and quadratic effects of increasing inclusion levels of AE in diets. Statistical significance and tendency were defined at *P* < 0.05 and 0.05 ≤ *P* < 0.10, respectively.

## Results and discussion

### Growth performance

Diets supplemented with 0.04% AE tended to increase (*P* = 0.070) BW and BWG than diets without AE (Table 2). Additionally, BW and BWG increased (linear, *P* < 0.05) as the inclusion levels of AE in diets increased. Birds fed diets containing 0.04% AE had greater (*P* < 0.05) FE than those fed a basal diet. In addition, FE also increased (linear, *P* < 0.05) as the inclusion levels of AE in diets increased. However, FI was not significantly different among the dietary treatments. Plants are known to contain a wide range of bioactive compounds, including alkaloids, steroids, tannins, glycosides, volatile oils, fixed oils, resins, phenols, and flavonoids, which are distributed variably among plant organs such as leaves, flowers, bark, seeds, fruits, and roots (Swelum et al., 2021). A variety of extraction techniques have been established to isolate these compounds, with aqueous (water-based) extraction recently gaining prominence due to its safety, eco-friendly attributes, and cost-effectiveness. This technique is well known for being especially suitable for isolating hydrophilic constituents such as flavonoids, phenolic acids, and polysaccharides, which are recognized for their antioxidant, anti-inflammatory, and immunomodulatory properties. In the current study, dietary AE supplementation resulted in improvements in BW, BWG, and FE as dietary inclusion levels of AE increased. *Abelmoschus* species have been identified as rich sources of quercetin. Quercetin, a polyphenolic compound derived from plants, is categorized as a flavonol, which is a subclass of flavonoids, and features a basic flavonoid structure comprising 15 carbon atoms arranged in 3 rings (C6-C3-C6). This compound is prevalent in a variety of fruits, vegetables, and herbs. In previous studies, Ying et al. (2022) reported that quercetin supplementation at 0.02, 0.04, or 0.06% not only enhanced antioxidant defenses but also activated immune-related pathways, supporting better growth performance in broiler chickens. In addition, Ahammad and Kim (2024) showed that micellar quercetin supplementation at 0.025, 0.050, or 0.100% exhibited significantly greater BWG compared with the control group. Given that AE is rich in quercetin, these findings suggest that quercetin may be one of the contributing bioactive compounds responsible for the enhanced growth performance. Furthermore, Abdel-Latif et al. (2021) demonstrated that dietary quercetin supplementation at 200, 400, or 800 ppm improved BWG and FE, crediting this to increased VH and upregulation of digestive enzyme genes in broiler chickens. It is well-known that an elevated VH:CD ratio correlates with enhanced nutrient absorption efficiency. In the present study, birds fed diets containing 0.04% AE exhibited significantly greater VH:CD ratio than those fed diets containing 0% and 0.02% AE. These morphological adaptations provide further evidence that dietary AE supplementation may facilitate nutrient uptake and digestion, which may explain the enhanced growth performance observed in broiler chickens. Collectively, these results reinforce the hypothesis that bioactive constituents in AE, especially quercetin, may contribute significantly to enhance growth outcomes in broiler chickens.

**Table 2:** Effect of dietary supplementation of *Abelmoschus manihot* L. flower extract (AE) on growth performance, liver antioxidant capacity, jejunal morphology, and welfare parameters in broiler chickens.1.

	Inclusion levels of AE in diets, %				*P-*value^2^		
Items	0	0.02	0.04	SEM	T	L	Q
Growth performance							
BW, g	1,695	1,769	1,890	58.1	0.070	0.023	0.725
BWG, g	1,652	1,725	1,847	58.1	0.070	0.023	0.725
FI, g	2,582	2,578	2,572	73.1	0.995	0.922	0.997
FE, g/kg	639^b^	670^b^	720^a^	15.9	0.005	0.001	0.608
Liver antioxidant capacity							
MDA, nmol/mg protein	0.43	0.64	0.57	0.119	0.447	0.408	0.338
TAC, nmol/mg protein	0.46	0.39	0.41	0.038	0.390	0.343	0.322
GSH, nmol/mg protein	0.15	0.10	0.22	0.047	0.213	0.277	0.152
CAT, U/mg protein	0.54	0.49	0.60	0.045	0.253	0.346	0.171
SOD, U/mg protein	1.23	1.26	1.34	0.096	0.660	0.382	0.825
Jejunal morphology							
Villus height, μm	1,069	1,042	1,088	54.3	0.814	0.803	0.560
Villus width, μm	153	137	126	8.5	0.104	0.037	0.811
Crypt depth, μm	142	132	117	7.5	0.099	0.035	0.787
VH:CD ratio	7.66^b^	7.91^b^	9.43^a^	0.467	0.030	0.016	0.243
VSA, mm^2^	0.52	0.46	0.43	0.045	0.400	0.195	0.740
Welfare parameters^3^							
Gait score	0.28^a^	0.21^a^^b^	0.06^b^	0.051	0.020	0.007	0.513
Footpad dermatitis	0.08	0.08	0.12	0.035	0.569	0.362	0.596
Hock burn	0.18	0.14	0.30	0.068	0.254	0.216	0.268
Plumage cleanliness	0.11	0.08	0.07	0.046	0.817	0.532	0.942

*Abbreviations*: BWG = BW gain; FI = feed intake; FE = feed efficiency (BWG:FI); MDA = malondialdehyde; TAC = total antioxidant capacity; GSH = glutathione; CAT = catalase; SOD = superoxide dismutase; VH:CD = villus height to crypt depth ratio; VSA = villus surface area.

a,b Means within a variable with no common superscript differ significantly (*P* < 0.05).

1 Data are least squares means of 8 observations per treatment.

2 T = overall effects of treatments; *L* = linear effects of increasing inclusion levels of *Abelmoschus manihot* L. flower extract in diets; *Q* = quadratic effects of increasing levels of *Abelmoschus manihot* L. flower extract in diets.

3 Gait score ranged from 0 to 5, with 0 representing normal gait and 5 indicating complete inability to stand or walk. Footpad dermatitis ranged from 0 to 4, with 0 indicating no lesion, 2 indicating mild lesion and very small superficial lesions, and 4 indicating severe lesion and signs of bleeding or swelling. Hock burn ranged from 0 to 4, with 0 indicating no inflammation, 1 to 2 indicating moderate inflammation, and 3 to 4 indicating advanced and widespread inflammation. Feather cleanliness ranged from 0 to 3, with 0 indicating clean and white feathers with absence of feces, and 3 indicating dirty brown feathers sometimes with feces adhered or caked to feathers.

### Liver antioxidant capacity

Dietary supplementation of AE resulted in no significant differences in liver antioxidant capacity among dietary treatments among dietary treatments (Table 2). It is well known that, under challenging conditions such as heat stress, toxin exposure, or environmental disturbances, phytogenic feed additives can enhance hepatic antioxidant defenses by promoting the activity of enzymes involved in oxidative balance and reducing lipid peroxidation. However, these widely reported responses were not observed in the present experiment, as dietary AE supplementation did not affect liver antioxidant capacity. This discrepancy may be associated with differences in experimental design and environmental conditions. In the present study, birds were raised under non-challenging, thermoneutral conditions, with no exposure to stressors capable of inducing oxidative stress. Therefore, the hepatic antioxidant defense system was likely sufficient to maintain redox balance, limiting the capacity of AE-derived bioactive compounds to exert additional antioxidant effects by further reducing lipid peroxidation.

### Jejunal morphology

The VW showed a linear decrease (*P* < 0.05) as the inclusion levels of AE in diets increased (Table 2). Birds fed diets containing 0.04% AE exhibited a tendency toward less (*P* = 0.099) CD compared to those fed a basal diet. Furthermore, CD decreased linearly (*P* < 0.05) with increasing inclusion levels of AE in diets. Diets supplemented with 0.04% AE resulted in a significantly greater VH:CD ratio (*P* < 0.05) than diets with 0 and 0.02% AE supplementation. The VH:CD ratio increased (linear, *P* < 0.05) as inclusion levels of AE in diets increased. However, VH and VSA were not significantly affected by dietary treatments. The integrity of the intestinal epithelium is a crucial indicator of intestinal health, as villus size, absorptive surface area, and the thickness of the mucus layer play are fundamental for transporting molecules and nutrients from the intestinal lumen into the bloodstream. In our experiment, as anticipated, increasing inclusion levels of AE in diets improved jejunal morphology. Structural improvements in the intestine are closely linked to better nutrient utilization and increased resistance to microbial invasion. In the current experiment, increasing inclusion levels of AE in diets increased VH:CD ratio, aligning with earlier results showing that birds fed diets with herbal feed additives at 0.5% or 1.0% exhibited enhanced intestinal morphology (Giannenas et al., 2018). These morphological changes are often linked to glucocorticoid levels, oxidative stress, and concentrations of inflammatory cytokines, regarding the proliferation of intestinal cells. Thus, the effect observed in this study may be due to the maintenance of mucosal integrity resulting from dietary AE supplementation, which may lessen stress responses and reduce apoptosis. Additionally, PFAs are known to promote gut health by influencing the composition of the intestinal microbiota. Wang et al. (2024) indicated that *Abelmoschus manihot* polysaccharide, administered to mice at 100, 200, or 400 ppm, improved the intestinal mucus barrier in mice by modulating *Akkermansia muciniphila*, a key symbiotic bacterium situated within the mucus layer. Therefore, we speculate that AE, which is highly bioavailable and rich in bioactive compounds, may produce positive effects on intestinal morphology. Collectively, these findings imply that AE may support improved intestinal structure by promoting mucosal integrity and alleviating stress-induced damage.

### Welfare parameters

In modern animal production systems, efforts to increase productivity through cage housing can adversely impact animal welfare. Cage environments frequently offer inadequate space, hinder the performance of natural behaviors, and result in environmental deficiencies for broiler chickens. Such restrictive conditions that limit physical activity have been linked to an increased prevalence of leg abnormalities in broiler chickens. This problem is especially evident in fast-growing broiler breeds, where the accelerated development of muscle tissue and BW imposes excessive mechanical stress on immature skeletal structures, joints, and ligaments. These welfare-related issues, including mortality and culling, ultimately diminish production efficiency and economic returns. Consequently, enhancing animal welfare through effective rearing management practices is critical for promoting normal behavior and optimizing growth performance in broiler chickens. In the present experiment, dietary AE supplementation led to improved gait scores in broiler chickens. Although the precise mechanism remains unclear, *Abelmoschus* species are rich in flavonoids and phenolic acids, which regulate inflammatory responses, possess free radical scavenging properties, and reduce cellular injury, thereby enhancing leg health and mobility in chickens (Kwon et al., 2022). Therefore, we propose that the beneficial effects of dietary AE supplementation on gait scores are primarily due to these bioactive compounds, thereby contributing to improved leg health in broiler chickens. To advance understanding in this field, further research is needed to clarify the underlying mechanisms by which dietary AE supplementation improves gait scores in broiler chickens reared in cage housing systems. In summary, dietary AE supplementation showed positive trends in growth performance and improvements in intestinal morphology without adverse effects on hepatic antioxidants in broiler chickens. Dietary AE also resulted in improved gait scores, suggesting no negative impact on welfare under the conditions of this study. Collectively, these findings may provide insights into the future application of AE as functional feed additive in broiler chickens and offer a potential nutritional strategy for improving growth performance and welfare-friendly rearing practices in modern poultry production. However, additional studies with more detailed physiological assessments and phytochemical analyses are required to further validate these effects and clarify the underlying mechanisms.

## Disclosures

The authors declare that they have no known competing financial interests or personal relationships that could have appeared to influence the work reported in the present study.

## CRediT authorship contribution statement

**Gyu Lim Yeom:** Writing – review & editing, Writing – original draft, Software, Methodology, Investigation, Formal analysis, Data curation, Conceptualization. **Ha Neul Lee:** Writing – review & editing, Software, Methodology, Investigation, Formal analysis, Data curation. **Yeong Bin Kim:** Writing – review & editing, Software, Methodology, Investigation, Formal analysis. **Ju Yeong Park:** Writing – review & editing, Software, Methodology, Investigation, Formal analysis. **Geun Yong Park:** Writing – review & editing, Software, Methodology, Investigation, Formal analysis, Conceptualization. **Ji Won Shin:** Writing – review & editing, Software, Methodology, Investigation, Formal analysis. **Sanghun Park:** Methodology, Investigation, Formal analysis. **Gyutae Park:** Methodology, Investigation, Formal analysis. **Soyoung Jang:** Methodology, Investigation, Formal analysis. **Yang-il Choi:** Writing – review & editing, Validation, Supervision, Resources, Project administration, Conceptualization. **Jungseok Choi:** Writing – review & editing, Validation, Supervision, Project administration, Methodology, Data curation, Conceptualization. **Jong Hyuk Kim:** Writing – review & editing, Writing – original draft, Validation, Supervision, Software, Project administration, Methodology, Investigation, Funding acquisition, Data curation, Conceptualization.
